# Evaluation of Delamination in Concrete by IE Testing Using Multi-Channel Elastic Wave Data

**DOI:** 10.3390/s20010201

**Published:** 2019-12-30

**Authors:** Seong-Hoon Kee, Jin-Wook Lee, Ma. Doreen Candelaria

**Affiliations:** 1 Department of Architectural Engineering, Dong-A University, 37 Nakdong-Daero 550 Beon-gil Saha-gu, Busan 69315, Korea; shkee0505@gmail.com (S.-H.K.); mecandelaria@up.edu.ph (M.D.C.); 2 Advanced Railroad Civil Engineering Division, Korea Railroad Research Institute, 176 Cheoldobangmulgwan-ro, Uiwang-si, Gyeonggi-do 16105, Korea

**Keywords:** multi-channel elastic wave measurement, delamination, concrete, lamb waves

## Abstract

The main objectives of this study are to develop a non-destructive test method for evaluating delamination defects in concrete by the Impact-echo test using multi-channel elastic wave data and to verify the validity of the proposed method by experimental studies in the laboratory. First, prototype equipment using an eight-channel linear sensor array was developed to perform elastic wave measurements on the surface of the concrete. In this study, three concrete slab specimens (1500 mm (width) by 1500 mm (length) by 300 mm (thickness)), with simulated delamination defects of various lateral dimensions and depth, were designed and constructed in the laboratory. Multi-channel elastic wave signals measured on the three concrete specimens were converted to the frequency-phase velocity image by using the phase-shift method. A data processing method was proposed to extract the dominant propagating waves and non-propagating waves from the dispersion images. The dominant wave modes were used to evaluate delamination defects in concrete. It was demonstrated that the surface wave velocity values were useful for characterizing the shallow delamination defects in concrete. In addition, the peak frequency of non-propagating wave modes extracted from the dispersion images gives information on the lateral dimensions and depths of the delamination defects. This study also discussed the feasibility of combined use of the results from propagating and non-propagating wave modes to better understand the information on delamination defects in concrete. As will be discussed, the multi-channel elastic wave measurements enable more accurate, consistent, and rapid measurements and data processing for evaluation of delamination defects in concrete than the single-channel sensing method.

## 1. Introduction

**Figure 1:**
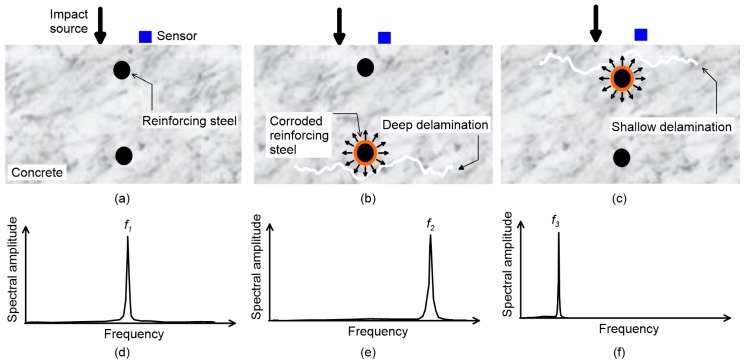
Typical source-and-receiver configuration and frequency signals of Impact-echo testing for condition assessment of concrete with delamination defects of various depths: the first column (**a**,**d**) represents solid (crack-free) concrete plates; and the second (**b**,**e**) and third (**c**,**f**) columns represent concrete plates with relatively deep and shallow delamination defects, respectively [5,6,7].

A delamination defect in concrete is a subsurface fracture plane that is caused by corrosion of embedded reinforcing steels in concrete (Figure 1b,c). The presence of delamination defects in concrete elements may not necessarily imply structural failure of reinforced concrete elements. However, various durability issues could arise by increasing moisture and/or deleterious materials in concrete, which could accelerate the corrosion and other deterioration mechanisms (i.e., freeze-thaw damage and carbonation). Furthermore, delamination defects in concrete with continuous corrosion can progress to open spalls and eventually degrade the structural integrity of reinforced concrete elements. Particularly, delamination defects in plate-like concrete elements in buildings and infrastructure systems (e.g., slabs in buildings, concrete bridge decks, and continuous concrete pavement) could critically affect the durability and/or structural integrity of concrete elements, which could threaten public users’ safety. Infrastructure management agencies in many countries have dedicated a great portion of the construction budget for maintenance of old/damaged concrete structures as opposed to new construction [1,2,3,4]. It is important to evaluate delamination in such a critical concrete element, and if necessary, to make an appropriate decision for the maintenance of concrete structures. As such, it will keep concrete elements in sound condition and enhance their service life. 

The impact-echo test has been proven to be an effective evaluation of delamination defects in plate-like concrete elements [5,6,7]. However, it was observed from preliminary studies in the laboratory and in the field tests that the conventional IE test that uses a single IE sensor has several limitations in its practical use. First, the use of single-channel measurement cannot provide velocity information of concrete. In many field applications of the IE method, the P wave velocity is determined from cores or from performing a test over the region of structure with known dimensions [8]. This approach, however, needs additional efforts associated with core extraction and/or performing wave velocity measurement with separate test equipment, which could be labor-intensive and could slow test speed. Furthermore, the P wave velocity values, estimated from several selected test regions with a separate test setup, may not represent either the velocity of concrete at the right location of the IE test or the velocity of entire concrete structures. Second, it was observed that an IE test using a single sensor has limitations in consistently measuring the S1 ZGV mode in the laboratory and field applications. This is mainly due to the interference of noisy signals that are not really associated with the mechanical properties of concrete [9]. The test results from single-channel measurements are not enough to separate important wave modes related to the critical features of delamination defects and solid concrete. Experiences from the laboratory and field survey show that noisy signals from unknown sources pose difficulties in signal interpretation of a single channel IE test. Third, the feature of the point-by-point method could limit the speed of the IE test [6]. IE method uses the non-propagating wave modes that are localized close to the impact source. Consequently, the single-channel IE test could be too slow to evaluate large concrete infrastructure systems. 

There are many kinds of research devoted to improving the conventional IE testing of concrete. Some researchers proposed a method to evaluate the pressure (P) wave velocity of concrete for determining the thickness of concrete by using the spectral analysis of surface waves (SASW) test [8]. In the said research, with assumed Poisson’s ratio, surface wave velocity estimated from SASW was converted to the P wave velocity of a test medium. Furthermore, some researchers developed a combination of IE testing with multi-channel elastic wave measurements [10]. In this research, the multi-channel analysis of surface waves (MASW) method was used to analyze both the propagating and non-propagating Lamb waves. It was proven to be effective in estimating the P wave velocity of concrete and to improve the detectability of the thickness stretch mode by using both the amplitude and phase information from the multi-channel record.

The primary purpose of this research is to develop an advanced IE test method for evaluating delamination defects in concrete using multi-channel elastic wave signal analysis. First, prototype equipment, supporting eight-channel elastic wave measurements, was developed and used to evaluate delamination defects in concrete specimens in the laboratory. Second, a signal processing method was proposed to extract important wave modes for the evaluation of delamination in the IE test. Third, the validity of the prototype equipment and data interpretation method was verified by comparison of experimental results. As will be discussed, the multi-channel elastic wave measurements enable more accurate, consistent, and rapid measurements and data processing for the evaluation of delamination defects in concrete compared to the single channel-sensing method.

## 2. Theoretical Background of IE

**Figure 2:**
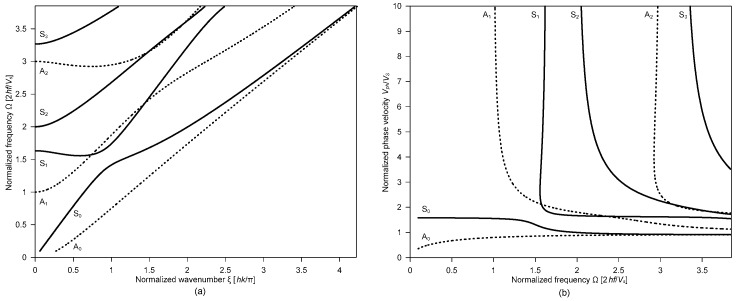
First several modes of Rayleigh–Lamb frequency equations: (**a**) normalized frequency and normalized wavenumber and (**b**) normalized phase velocity and normalized frequency [16,17].

There have been many prior researches that used dispersive behavior of elastic waves for non-destructive evaluation (NDE) of various structural elements [11,12,13,14,15]. The IE test is an NDE method that uses dispersion of transient elastic waves for evaluation delamination defects in plate-like concrete structures. It is based on dynamic response measurements of concrete over delamination defects, in which an impact point source is used to generate incident waves. Figure 1 shows the typical test setup of IE method over delamination defects with various depths in a concrete plate. The mechanical impact, applied on the surface of the concrete, generates body waves (pressure (P) and shear (S) waves) and surface-guided waves (e.g., Rayleigh surface waves) that propagate in the concrete plate (see Figure 1). The body waves, that were reflected and mode-converted multiple times, eventually built up sets of infinite wave modes [16,17], that can be determined from Rayleigh–Lamb frequency equations as follows:

(1) $$\frac{\tan\left[\frac{1}{2}\pi {\left(\Omega^{2}-\xi^{2}\right)}^{\frac{1}{2}}\right]}{\tan\left[\frac{1}{2}\pi {\left(\Omega^{2}/\kappa_{0}^{2}-\xi^{2}\right)}^{\frac{1}{2}}\right]}=-\frac{4\xi^{2}{\left(\Omega^{2}/\kappa_{0}^{2}-\xi^{2}\right)}^{\frac{1}{2}}{\left(\Omega^{2}-\xi^{2}\right)}^{\frac{1}{2}}}{{\left(\Omega^{2}-2\xi^{2}\right)}^{2}}\;\mathrm{for}\;\mathrm{Symmetric}\;\mathrm{modes}$$

(2) $$\frac{\tan\left[\frac{1}{2}\pi {\left(\Omega^{2}-\xi^{2}\right)}^{\frac{1}{2}}\right]}{\tan\left[\frac{1}{2}\pi {\left(\Omega^{2}/\kappa_{0}^{2}-\xi^{2}\right)}^{\frac{1}{2}}\right]}=-\frac{{\left(\Omega^{2}-2\xi^{2}\right)}^{2}}{4\xi^{2}{\left(\Omega^{2}/\kappa_{0}^{2}-\xi^{2}\right)}^{\frac{1}{2}}{\left(\Omega^{2}-\xi^{2}\right)}^{\frac{1}{2}}}\;\mathrm{for}\;\mathrm{Asymmetric}\;\mathrm{modes}$$

where

$$\Omega =\frac{2hf}{C_{s}},\;\xi =\frac{hk}{\pi }\;\mathrm{and}\;\kappa_{0}=\sqrt{\frac{2\left(1-\nu \right)}{1-2\nu }}$$

h = plate thickness, f = frequency, k = wave number and $C_{s}$ = shear wave velocity. Figure 2a shows first several Lamb wave modes for concrete with Poisson’s ratio of 0.22, which is presented in the normalized frequency (Ω = 2 hf/C_s_) and normalized wave number (*ξ* = hk/*π*) domain. The dynamic response of concrete over delamination defects is generally measured by a sensor located closed to the impact source (about 30 to 50 mm). The Fourier transform of the time signal shows peaks at certain frequencies, which are interpreted as dominant non-propagating wave modes (or resonance modes).

Two sets of resonance modes associated with Lamb waves in a plate are generally used to detect and characterize delamination defects in concrete using IE testing, which includes the thickness stretch mode and the flexural vibration mode [6]. The frequency analysis of the two resonance modes provides useful information on the presence and dimensions of delamination defects (areal extensions and depths) in concrete. First, the thickness stretch mode dominates the spectral response of a plate that does not contain any near-surface defects (see Figure 1). Theoretically, thickness stretch mode results from multiple reflections of *P* waves between the top and bottom surfaces of a solid plate. The resonance frequency of the thickness stretch mode is the cut-off frequency of the first symmetric Lamb mode [18]. However, it has been observed that the dominant frequency from the conventional IE test setup is slightly lower than the thickness stretch mode [19,20]. The resonance frequency from the IE test was related to the zero-group velocity of the first symmetric Lamb mode (S1-ZGV frequency, *fs*_1*ZGV*_). The S1-ZGV frequency can be used to determine the thickness, h, of a crack-free plate when *P* wave, *C_p_*, the velocity of concrete is known by

(3) $$h=\frac{\beta C_{P}}{2f_{S1ZGV}},$$

where *β* is a correction factor that depends on Poisson’s ratio of concrete [19], and is in the range of 0.945 to 0.957 for concrete with Poisson’s ratio from 0.16 to 0.25. Furthermore, some researchers noticed that the dominant resonance frequency from IE testing of actual materials with some damping capacity is slightly higher than the S1-ZGV frequency obtained from the Rayleigh-Lamb frequency equation (Equation (1)). The actual resonance mode results from the interference of two non-propagating Lamb modes (S1 and S2b) that have very close phase velocities at just above the S1-ZGV frequency, where S2b is “backward wave” of the second symmetric Lamb mode [21,22,23]. Nonetheless, Equation (1) is still valid and useful to relate the dominant frequency from the IE test and the thickness of a test plate since there is only a little difference between *f_S_*_1_ and *f_S_*_2*b*_. In this study, concrete with relatively deep delamination with a depth of 250 mm was also characterized by an analysis of the S1-ZGV mode. Given *f_S_*_1*ZGV*_ and *C_p_*, the depth of the relatively deep delamination can be estimated reliably using Equation (3). In contrast, relatively shallow delamination defects could be characterized by flexural vibration modes. Flexural vibration modes can be interpreted as non-propagating waves as a consequence of multiple reflections of Lamb waves at delamination [24]. It was observed that the propagating Lamb waves (A0 and S0) over a delamination defect were reflected and transmitted multiple times at the edges of the delamination defect. The resonance frequency of the flexural vibration mode, $f_{flex}$, is dependent on the plate geometry (lateral dimensions and depth), shape, and boundary conditions [25,26]. The analysis of $f_{flex}$ is effective for evaluating the presence of relatively shallow delamination defects in concrete and for estimating the areal extents of the shallow defects [26]. 

## 3. Experiments

### 3.1. Preparation of Concrete Specimens

**Figure 3:**
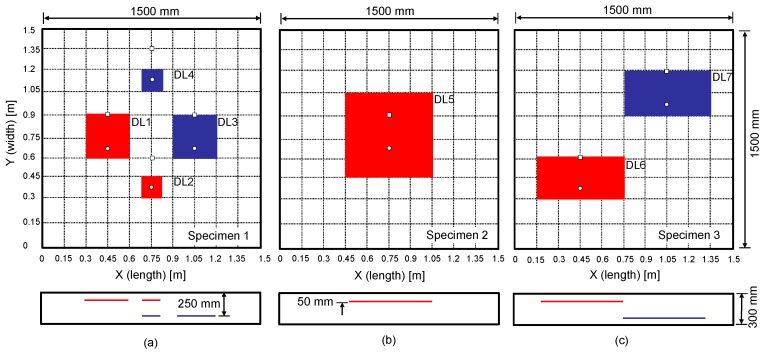
Concrete specimens with simulated delamination defects of various sizes and depths: (**a**–**c**) for plan views of specimen 1, specimen 2, and specimen 3 with horizontal and vertical distribution of delamination defects, respectively.

**Figure 4:**
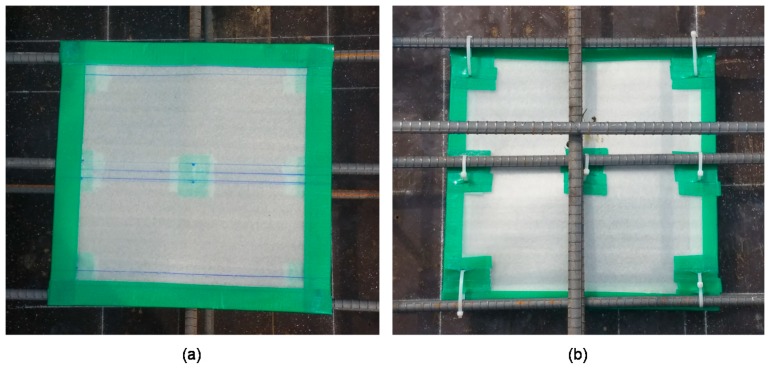
Simulated delamination defects which were attached to reinforcing steel mat before casting concrete: (**a**) DL 1 and (**b**) DL 3 for simulating shallow and deep delamination defects, respectively.

Three concrete slab specimens were fabricated at Dong-A University, as part of a research project to develop an innovative condition assessment method for old or damaged urban subway concrete tracks in Korea. The concrete specimens were designed to have dimensions of 1500 mm (length) × 1500 mm (width) × 300 mm (depth) (see Figure 3), which is large enough to simulate a full-scale traffic control layer (TCL) in urban subway concrete tracks in the country. In addition, the concrete slabs were built with two mats of uncoated steel reinforcement at 50 and 250 mm depths, respectively. Each of the reinforcement mats consists of D13 steel bars with a diameter of 13 mm, spaced at 300 mm in both directions. Concrete was made of Type 1 Portland cement (440 kg/m^3^), river sand (701 kg/m^3^), crushed coarse aggregate (1049 kg/m^3^), and water (165 kg/m^3^). The mixture was designed to have a minimum 28-day compressive strength of 35 MPa. The concrete slabs were water-cured for seven days after casting. The actual measured 28-day strength was higher than the design with a value of 37.5 MPa. The assembled concrete slabs were designed to contain simulated delamination defects using double-layered thin film with a thickness of 50 µm of various sizes and at two different depths (see Figure 4): shallow delamination defects at 50 mm depth and deep delamination defects at 250 mm depth. Figure 3 shows an overview of the horizontal and vertical distribution of delamination as built in the concrete slab.

### 3.2. Test Setup for Multi-Channel Elastic Wave Measurements 

**Figure 5:**
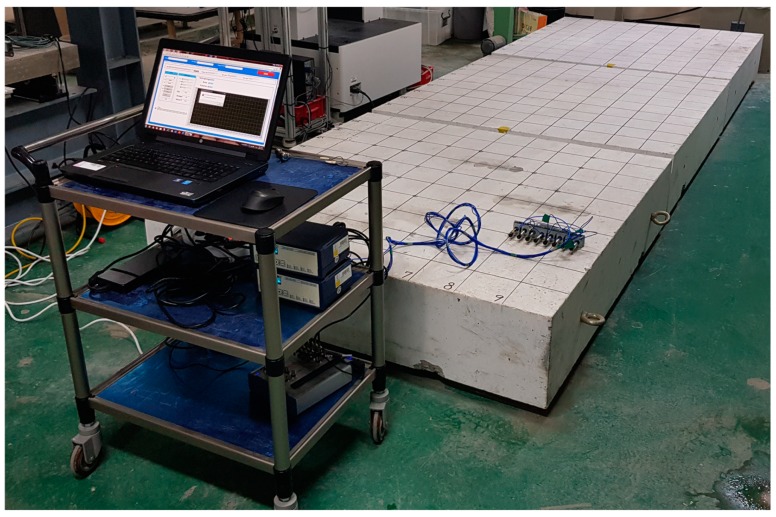
A prototype equipment for multi-channel elastic wave measurements on concrete specimens.

**Figure 6:**
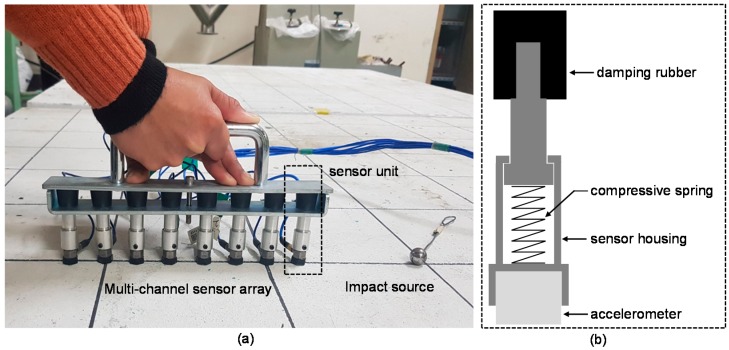
(**a**) A photo of elastic wave measurements on the surface of concrete using the multi-channel sensor array developed in this study and (**b**) a sensing unit composed of an accelerometer, a sensor housing, a compressive spring and a damping rubber.

Prototype equipment for multi-channel elastic wave measurements on the surface of the concrete slab, shown in Figure 5, was produced by a research team at Dong-A University. The prototype equipment is composed of a multi-channel sensor array with eight sensing units, signal conditioners, a data acquisition system, and a laptop computer. A sensing unit includes an accelerometer, a sensor housing, and a damping rubber. The accelerometer in the sensing unit (Model: PCB 352C33) has a diameter of 15.7 mm, broad bandwidth (0.5 Hz to 10 kHz), high resonance frequency (≥50 kHz), and high sensitivity (10.2 mV/(m/s^2^)) [27]. A sensor housing was developed to hold an accelerometer during multi-channel elastic wave measurements (see Figure 6b). It contains a compressive spring that exerts pressure on the sensor when it is pushed. It was observed that the pressure by the compressive spring was effective in providing reliable coupling of the sensor to the surface of concrete without applying additional coupling agents. The sensor housing was inserted into a damping rubber to reduce the interference of noisy signals with useful signals associated with the dynamic response of concrete. The use of multi-channel sensors and the dry contact technique significantly improves the test speed of elastic wave measurements on concrete. A steel ball with a diameter of 15 mm was used as an impact source. The concrete slab vibrations built up by the impact source were measured by eight accelerometers and amplified by two signal conditioners (480E09). The output signals were digitized at a sampling frequency of 500 kHz using a NI-USB 5132 oscilloscope. Each dynamic signal was collected for a duration of 10 ms. The acquired signals were transferred to and stored in a laptop computer. The data acquisition and saving procedures were controlled by a LabVIEW-based computer program. All equipment was carried on a cart and was operated by two persons. 

### 3.3. Test Procedure

**Figure 7:**
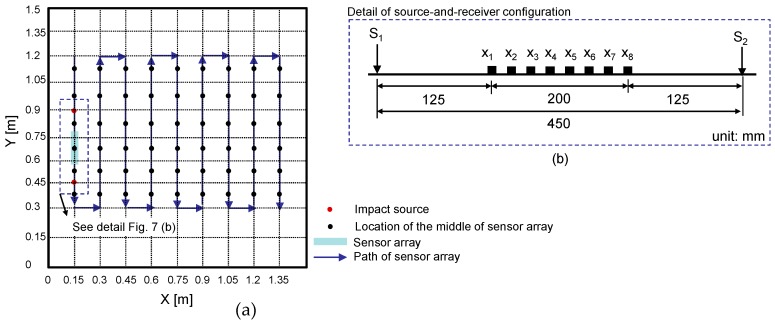
Test plan on the surface of a concrete specimen: (**a**) scanning procedure for multi-channel elastic wave measurements and (**b**) source-and-receiver configuration.

The prototype equipment was used to measure dynamic responses of test regions on the three concrete specimens. Each measurement using the eight-channel sensor array covers a test line of 200 mm long. In this study, the sensor array was kept parallel to the vertical axis with the middle of the sensor at each test point, which is presented as a black solid circle in Figure 7a. Figure 7b shows the source-and-receiver configuration for multi-channel elastic measurements in a test region. At each test point, an impact source was placed on either side of the test region. First, incident elastic waves were generated by an impact loading applied at the location of S1, which is on the left side of the sensor array and was measured by the eight sensors simultaneously. Next, an independent test was repeated by applying an impact loading at S2, which is on the right side of the sensor array. The elastic wave measurement was repeatedly performed in the transverse direction (see Figure 7a). The data scans represented a total of 54 test regions on the surface of each concrete specimen.

## 4. Analysis of Multi-Channel Elastic Wave Signals

### 4.1. Typical Time Signals 

**Figure 8:**
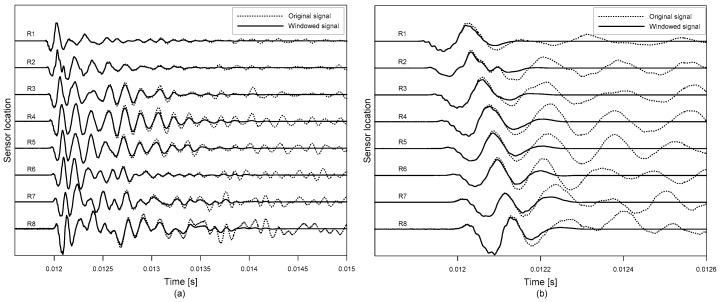
Typical time signals measured by the eight-channel sensor array after applying (**a**) a broad and (**b**) a narrow window function, respectively.

Figure 8 shows typical time signals, u(r,s,t), measured at the test point, *r* = ($x_{r},y_{r})$ = (1.35 m, 0.975 m) and *s* = ($x_{s},y_{s})$ = (1.35 m, 1.2 m), on the concrete specimen 1; where $x_{r}$ and $y_{r}$ are location of the middle of the sensor array; $x_{s}$ and $y_{s}$ represent the location of the impact source, and *t* is time. In this study, two sets of windowed signals were obtained by applying Hanning windows with two different sizes (i.e., 20 times and 2 times of period) at the minima of first dominant time wave signals. Figure 8a,b show the broad and narrow windowed signals, $u_{bw}^{}\left(r,s,t\right)$ and $u_{nw}^{}\left(r,s,t\right)$, respectively. It was confirmed that $u_{bw}^{}\left(r,s,t\right)$ and $u_{nw}^{}\left(r,s,t\right)$ are effective for extracting resonance modes (flexural vibration mode over shallow delamination and/or thickness stretch mode over solid concrete or relative deep delamination) and propagating Lamb waves, such as A0 Lamb wave mode, respectively. 

### 4.2. Dispersion Imaging Scheme by the Phase-Shift Method 

**Figure 9:**
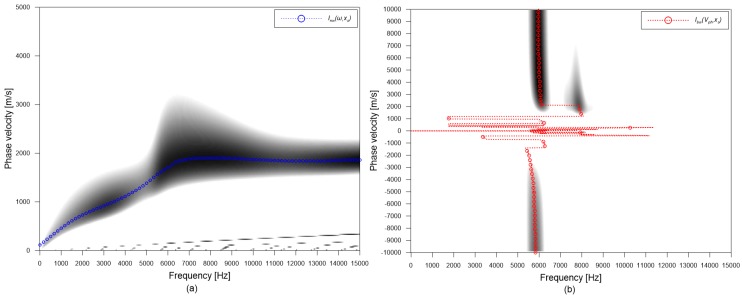
Typical dispersion images created by the plane wave transformation method for (**a**) narrow and (**b**) broad windowed time signals.

The windowed space and time domain signals, $u_{j}^{}\left(r,s,t\right)$, measured using multi-channel elastic wave measurements by hitting an impact source located at $x_{s}$, were transformed to the frequency and phase velocity domain, $S_{j}\left(\omega ,V_{ph},x_{s}\right)$, using the phase-shift method [28] as follows,

(4) $$S_{j}\left(\omega ,V_{ph},x_{s}\right)=\int e^{-i\left(\frac{\omega }{V_{ph}}\right)x_{r}}[U_{j}\left(r,s,\omega \right)/\left|U_{j}\left(r,s,\omega \right)\right|]dx_{r}$$

where $U_{j}\left(r,s,\omega \right)$ is the Fourier transformation of $u_{j}^{}\left(r,s,t\right)$; where *j* represents the type of windowed signals (*bw* and *nw* for the broad and narrow windowed signals, respectively), r is location of sensors, s is location of the impact source, *t* is time, ω is the angular frequency (2πf), $V_{ph}$ is phase velocity. The integral results in a value in the range of 0 to 1 for a set of frequency and phase velocity. Figure 9a,b show the typical dispersion images created from the windowed time signals with narrow and broad windowing functions, respectively. In this study, a dominant propagating wave mode was extracted from the dispersion image of narrow windowed time signals by finding a peak amplitude along the vertical axis for a given frequency as follows,

(5) $$I_{nw}^{}\left(\omega ,x_{s}\right)=\underset{V_{ph}}{\arg\max}S_{nw}\left(\omega ,V_{ph},x_{s}\right)$$

A resulting dispersion curve, $I_{nw}^{}\left(\omega ,x_{s}\right)$, is presented as a blue dash line with open circles in Figure 9a, which appears to match with A0 Lamb mode. Furthermore, dominant non-propagating waves were extracted by finding a peak amplitude along the horizontal axis for a given phase velocity as follows,

(6) $$I_{bw}^{}\left(V_{ph},x_{s}\right)=\underset{\omega }{\arg\max}S_{bw}\left(\omega ,V_{ph},x_{s}\right)$$

**Figure 10:**
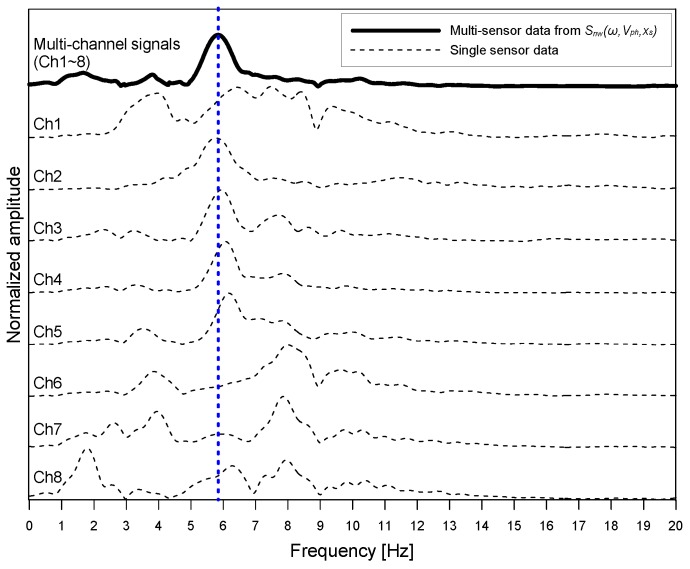
Comparison of spectral amplitude obtained from multi-channel data and single-channel data.

A resulting dispersion curve, $I_{bw}^{}\left(V_{ph},x_{s}\right)$, is shown as a red dash line with open circles in Figure 9b. Dominant resonance waves have various phase velocities ranging from 2000 m/s to 10,000 m/s and from −2000 m/s to −10,000 m/s within a narrow frequency range between 5.5 kHz and 6.5 kHz. However, it was difficult to indicate the S1 ZGV point from the extracted dispersion curve. For this study, the amplitude of dispersion image was summed over the negative velocity range (for the backward wave) for each frequency to determine the frequency of dominant waves [23], in accordance with the Lamb wave theory of solid materials with damping [22,29] (see Figure 10). The spectral amplitude obtained from the dispersion image shows a clear peak at 5.8 kHz that results in an estimated thickness of 293 mm by Equation (1). For comparison, spectral amplitude obtained by the Fourier transform of single-channel time signals are shown in Figure 10. It was observed that multiple spectral peaks pose difficulties in the correct interpretation of data from an IE test.

### 4.3. Analysis of Non-Propagating Waves over Delamination Defects

**Figure 11:**
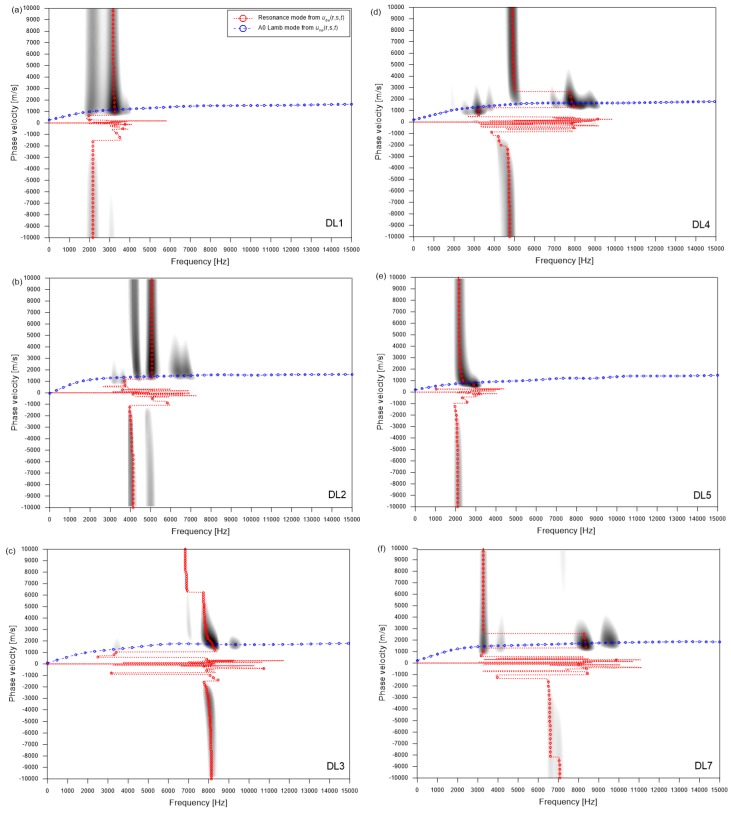
Frequency-phase velocity images created by the plane wave transformation method using multi-channel signals obtained over various delamination defects in concrete specimens: (**a**) DL 1, (**b**) DL2, (**c**) DL3, (**d**) DL4, (**e**) DL5, and (**f**) DL7.

Figure 11 shows typical dispersion images resulting from multi-channel elastic wave measurements over the simulated delamination defects (DL1~5 and DL7) in the three concrete specimens. The dispersion image for DL6 was like that of DL1 and was not shown in this article. The locations of the sensor array and impact source used to obtain the results shown in Figure 11 were presented as white solid circles and squares, respectively, in Figure 3. Dispersion curves of dominant non-propagating waves (the thickness stretch mode and/or flexural vibration mode) and propagating waves (A0 Lamb mode) are shown as red and blue dash lines, respectively, in Figure 11. It was recognized that the presence of delamination defects with different lateral dimensions and depths results in the variation of dispersion curves of dominant waves. 

**Figure 12:**
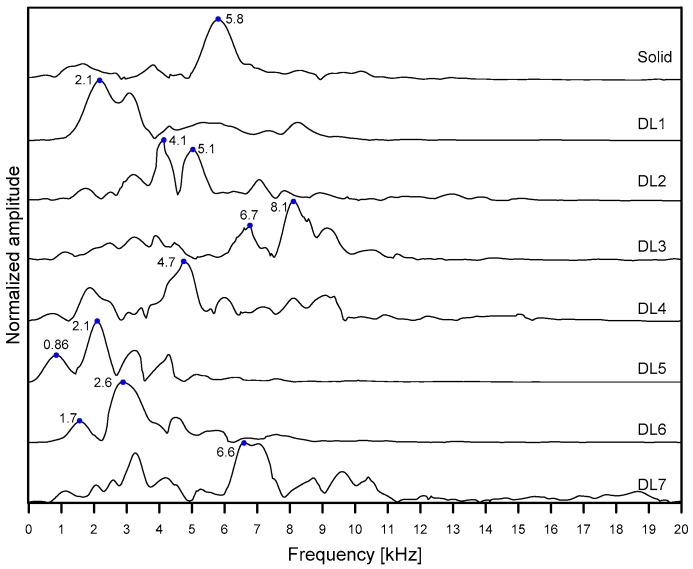
Normalized spectral amplitude obtained by summing the amplitude of the dispersion images for each frequency.

Figure 12 is a plot representing the variation of spectral amplitude created by summing the amplitude of the dispersion images over a negative velocity range from −5000 m/s to −10,000 m/s for each frequency. The peak frequencies of non-propagating waves are shown in Figure 12 and summarized in Table 1. Deep delamination defects with relatively large lateral dimensions (DL3 and DL7) result in a relatively high-frequency response (6.6 kHz and 6.7 kHz, respectively) than that over solid concrete (5.8 kHz). The estimated depths evaluated by Equation (1) were about 260 and 257 mm, respectively, which are close to the as-built depth (250 mm) of the deep delamination defects in this study. Therefore, the peak frequencies obtained from the dispersion images (Figure 11c,f) can be interpreted as the thickness stretch mode of the deep delamination defects. However, consistent with previous observation [6], it was observed that the size of delamination defects has a great influence on the estimation error of the depth of delamination defects using the analysis of the S1 ZGV Lamb mode (e_dS1ZGV_) (see Table 1). A deep delamination defect with small lateral dimensions (DL4) exhibits a peak frequency of about 4.7 kHz, which is lower than the thickness stretch mode frequency over solid concrete: thus, it does not result in accurate depth estimation. Lateral dimensions should be at least 300 mm to set up the S1 ZGV Lamb mode and to obtain relatively accurate depth estimation.

In contrast, the shallow delamination defects (DL1, DL2, DL5, and DL7) resulted in the low peak frequency values equal to about 2.1 kHz, 4.1 kHz, 2.1 kHz, and 2.6 kHz, respectively. These values were lower than that of the thickness stretch mode measured on the solid concrete. As seen in Table 1, the estimated depths based on the peak frequencies significantly overestimated compared to the as-built depths, with estimation errors of 1470%, 742%, 1544%, and 1402%, respectively. The low peak frequency values from the relatively shallow delamination defects are related to the flexural vibration mode over the delamination defects. For the shallow delamination defect with relatively large lateral dimensions, DL5, several resonance modes were observed at the peak frequencies of about 0.86 kHz, 2.1 kHz, 3.23 kHz, and 4.26 kHz, which appears to be the fundamental and higher-order modes of flexural vibration modes. It was demonstrated by the previous study [26] that the analysis of flexural vibration modes over shallow delamination defects in concrete is effective for estimating the lateral dimensions of the defects. In this study, lateral dimensions of shallow delamination defects (DL1, DL2, DL5, and DL7) were estimated by an approximate equation relating to the fundamental frequency of flexural vibration and the dimensions of delamination defects suggested in previous research [26]. The estimated lateral dimensions, shown in Table 1, have a good agreement with the smaller width of the as-built lateral dimensions, which supports the theory that the low frequency resonance is associated with the flexural vibration modes over shallow delamination defects. The estimation errors (e_c_) seem to be in an acceptable level (from 4.6% to 13%, shown in Table 1), without regard to the size of delamination defects considered in this study.

### 4.4. Analysis of Propagating Waves over Delamination Defects

**Figure 13:**
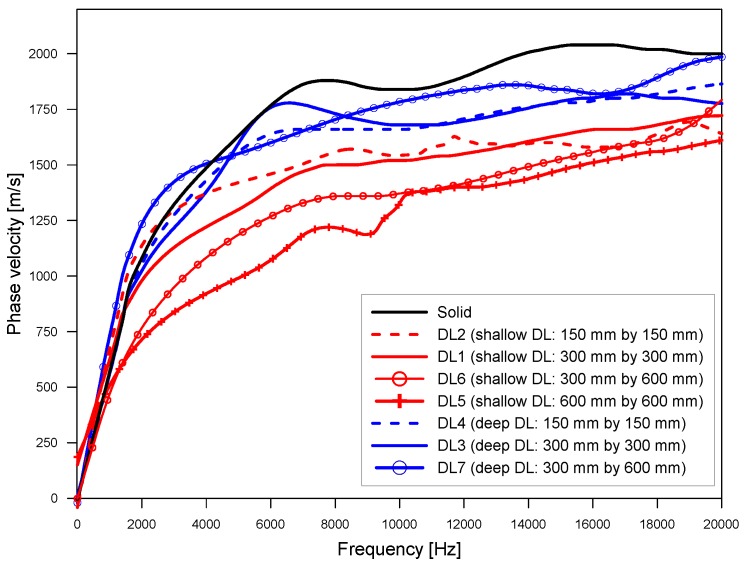
The variation of dispersion curves of A0 Lamb mode over delamination defects with various lateral dimensions and depths.

Figure 13 shows the variation of dispersion curves representing dominant propagating wave mode (A0 Lamb mode) over the delamination defects DL1~DL7 and solid concrete. The locations of the sensor array and impact source used to obtain the results shown in Figure 13 were presented as white solid circles and squares, respectively, in Figure 3. The presence of delamination in concrete affects the propagation of A0 Lamb mode. In this study, two critical parameters were extracted from the dispersion curves of the A0 Lamb modes: surface wave velocity and depths of delamination defects.

Theoretically, the phase velocity of A0 Lamb wave mode gradually increases from zero to the surface wave velocity, V_R_, as the frequency increases from zero to infinity. In this study, the surface wave velocity of concrete was evaluated by averaging the phase velocity of A0 Lamb mode in a frequency range from 10 kHz to 15 kHz. Resulting surface wave velocity over delamination defects are summarized in Table 1.

In addition, the depth of shallow delamination defects was estimated by finding an optimum analytic model that results in the least RMSE (root mean square error) compared to the experimental curve as follows,

(7) $$RMSE\left(h\right)=\sqrt{\frac{1}{N}\sum_{i=1}^{N}{\left(\Psi_{i}-\Psi_{i}^{\prime }\right)}^{2}}$$

where $\Psi_{i}$ is the normalized phase velocity (phase velocity normalized by shear wave velocity) obtained from the experimental data corresponding to $\Omega_{i}$, *i* is the index of experimental data, *N* is the number of data, $\Psi_{i}^{\prime }$ is the normalized phase velocity of the analytic model. In this study, an approximate formula relating Ψ and Ω was established by non-linear regression analysis of the Rayleigh-Lamb frequency equation for a fixed Poisson’s ratio of 0.2 as follows,

(8) $$\Psi_{i}^{\prime }=\frac{\sum_{i=0}^{4}a_{i}\Omega^{i}}{\sum_{i=0}^{4}b_{i}\Omega^{i}}$$

where *a*_0_ = 3.3, *a*_1_ = 2026, *a*_2_ = 2492, *a*_3_ = 6494, *a*_4_ = −45.91, *b*_0_ = 291.9, *b*_1_ = 3378, *b*_2_ = 3337, *b*_3_ = 6714, and *b*_4_ = 1. Table 1 also summarizes the estimated depth of delamination. It was observed that the sensitivity of the $\Psi_{i}^{\prime }$(h) gradually decreases as the depth of delamination defects increases. In this study, the estimated depth from the A0 Lamb wave is valid for relatively shallow delamination (i.e., the delamination depth is less than 100 mm). Table 1 summarizes the estimation errors of the depth of relatively shallow delamination defects (e_dA0_) by the analysis of the A0 Lamb wave mode. The estimation error (e_dA0_) increases as the lateral dimension decreases. In particular, the estimated depth of DL2 with a relatively smaller width (150 mm) was significantly overestimated compared to the as-built size, with an estimation error of 112%. Therefore, lateral dimensions should be sufficiently large (at least 300 mm) to obtain relatively accurate depth estimation by the A0 Lamb wave mode analysis. 

## 5. Visualization of Delamination Defects in Concrete

Based on the observations in the previous sections, delamination defects in concrete were visualized by mapping the three critical parameters, which include surface wave velocity, peak frequency, and depth of delamination defects. The three delamination maps were constructed based on 100 by 100 grid points. The data values between the measured data points (a total of 6 by 9 points) were obtained by interpolation using the ‘kriging algorithm’. The maps were created by a commercially available mapping program, Surfer [30].

### 5.1. Surface Wave Velocity Map

**Figure 14:**
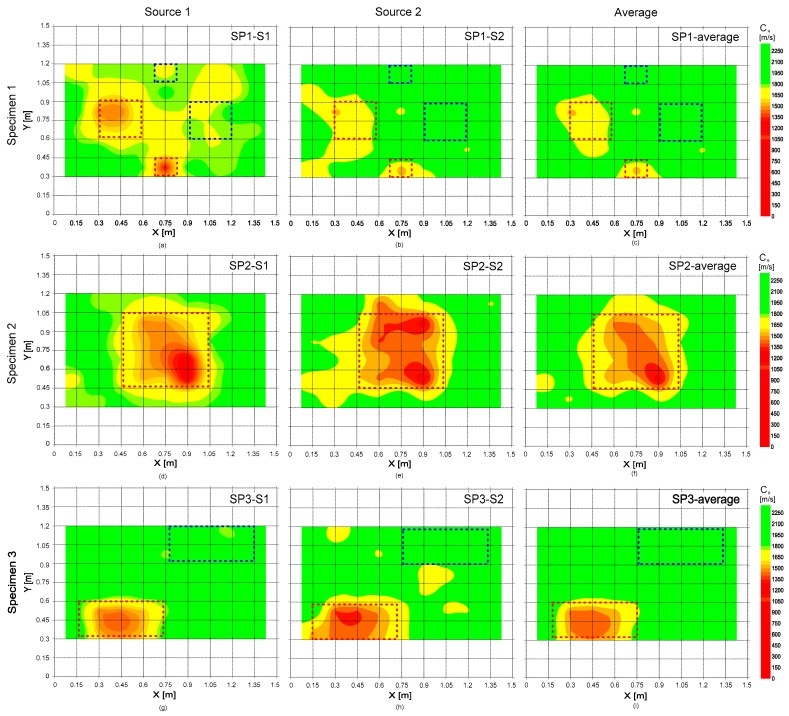
The delamination map representing the distribution of surface wave velocity values measured on the three concrete specimens. The figures in the first (**a**–**c**), second (**d**–**f**) and third rows (**g**–**i**) represent the results from the concrete specimens 1, 2, and 3, respectively. The first (**a**,**d**,**g**) and second (**b**,**e**,**h**) columns represent the results obtained from the signals generated by the impact source 1 and 2, respectively; and the average of the two results are shown in the third column (**c**,**f**,**i**).

Figure 14 shows the surface wave velocity map representing the variation of surface wave velocity obtained from the analysis of A0 Lamb mode which was measured on the surface of the three concrete specimens. The figures in the first, second, and third rows represent the results from the concrete specimens 1, 2, and 3, respectively. The first and second columns represent the results obtained from the signals generated by the impact source 1 and 2, respectively; and the average of the two results are shown in the third column. The deep delamination defects (DL3, DL4, and DL7) were not clearly differentiated from the solid concrete. In contrast, all shallow delamination defects (DL1, DL2, DL5, and DL6) in this study were clearly identified as low surface velocity. The lower phase velocity over the shallow delamination defects is mainly caused by the dispersion of propagating waves trapped between ‘Entrance’ and ‘Exit’ [24]. 

Furthermore, a statistical analysis was performed to confirm the effectivity of the surface wave velocity values as critical parameters for evaluating delamination defects in concrete. Test regions on the three concrete specimens were classified into three groups: ‘Group 1’ over solid concrete, ‘Group 2’ over shallow delamination defects, and ‘Group 3’ over deep delamination defects. The surface wave velocity data in the three groups follow the normal distribution, which was checked by the Kolmogorov-Smirnov (K-S) test [31]. Table 2 summarizes the results of the K-S test with the mean, standard deviation, and coefficient of variation (COV) of the surface wave velocity values in the three data groups. It was observed that there is a significant difference between the means of Group 1 and 3 at the 5% significance level according to the *t*-test. This statistical result indicates that surface wave velocity could be a useful parameter to identify the concrete with shallow delamination defects from solid concrete. However, no significant difference was found between Group 1 and Group 2. Still, it should be noted that there are several reasons explaining the decrease in the surface wave velocity of concrete (e.g., reduced elastic modulus due to degradation materials) in actual concrete structures [2,32]. Therefore, the lower surface wave velocity may not necessarily imply the presence of shallow delamination defects in actual concrete elements in the field. Nevertheless, the observation of this study could enhance the knowledge explaining one reason for lower surface wave velocity in the field.

### 5.2. Frequency Map

**Figure 15:**
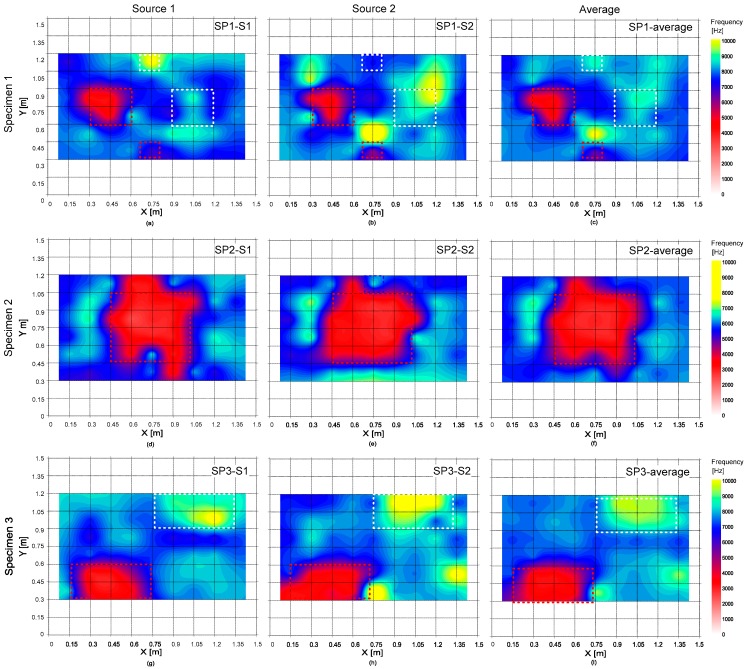
The delamination map representing the distribution of peak frequency values measured on the three concrete specimens. The figures in the first (**a**–**c**), second (**d**–**f**) and third rows (**g**–**i**) represent the results from the concrete specimens 1, 2, and 3, respectively. The first (**a**,**d**,**g**) and second (**b**,**e**,**h**) columns represent the results obtained from the signals generated by the impact source 1 and 2, respectively; and the average of the two results are shown in the third column (**c**,**f**,**i**).

Figure 15 shows the frequency map representing the variation of the peak frequency on the three concrete specimens. The first and second columns represent the frequency maps obtained from the signals generated by the impact source 1 and 2, respectively, and the average of the two frequency values in the first and second columns for each test specimen is shown in the third column. Overall, the frequency map appears to be effective for differentiating the locations of delamination defects with different sizes and depths from the solid concrete regions. 

**Figure 16:**
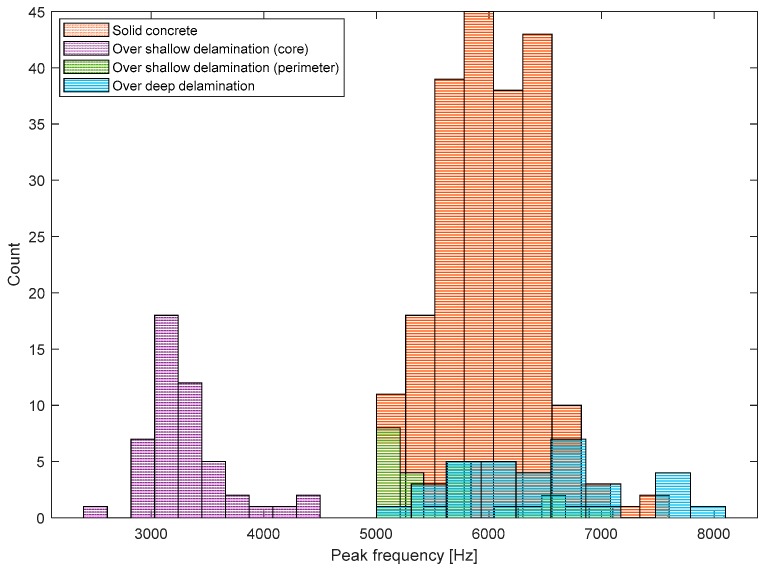
Histograms representing the distribution of peak frequency values measured on the three test regions 1, 2, and 3, each of which represents the test region over solid concrete and deep and shallow delamination defects.

Figure 16 shows histograms representing the distribution of peak frequency values measured on the three test regions 1, 2 and 3, each of which represents the test region over solid concrete and deep and shallow delamination defects. According to the Kolmogorov–Smirnov test, the peak frequency data on the test regions 1 and 2 follow the normal distribution (see Table 3). However, two peaks were clearly observed in the histogram of the peak frequency data obtained from test region 3 (over shallow delamination defects). Two peaks in the histogram indicate that the resulting data may come from two different sources. In this study, the frequency data from test region 3 was further divided into two groups according to the threshold frequency value of 5000 Hz. It was observed that peak frequency data higher than 5000 Hz were obtained from around the perimeter of the shallow delamination defects. Previous researchers discussed the difficulties in signal interpretation of IE test data obtained around the perimeter of the shallow delamination defects [2,33]. In this study, only the frequency data lower than 5000 Hz was used to characterize the shallow delamination defects. The Kolmogorov–Smirnov test shows that low-frequency data from test region 3 follows the normal distribution. Table 3 summarizes the mean and standard deviation of frequency values in four groups (solid concrete, deep delamination defect, low and high-frequency data from shallow delamination defects). According to a series of *t*-tests, there is a significant difference between the means of Group 1 and 3 and Group 1 and 2 at the 5% significance level.

### 5.3. Thickness Estimation 

**Figure 17:**
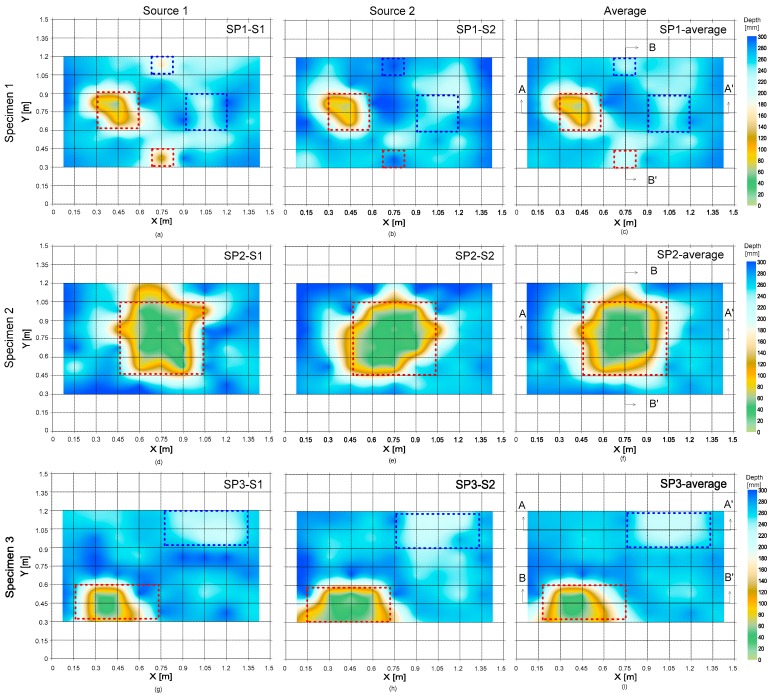
The delamination map representing the depths of delamination defects in the three concrete specimens. The figures in the first (**a**–**c**), second (**d**–**f**) and third rows (**g**–**i**) represent the results from the concrete specimens 1, 2, and 3, respectively. The first (**a**,**d**,**g**) and second (**b**,**e**,**h**) columns represent the results obtained from the signals generated by the impact source 1 and 2, respectively; and the average of the two results are shown in the third column (**c**,**f**,**i**).

It is of interest to engineers in infrastructure management agencies to estimate the depth of delamination defects and the thickness of concrete structures. Figure 17 shows the delamination maps representing the areal extension and the depth of delamination defects. In this study, the depth information of the delamination defects was estimated by a rule-based approach using the combination of the A0 Lamb wave analysis and the dominant frequency of S1 ZGV mode. In this study, the thickness of solid concrete (or depth of the delamination defects) was calculated by Equation (3), when the thickness estimated from the A0 Lamb wave analysis is greater than 100 mm. Otherwise, the depth was estimated based on the A0 Lamb analysis.

**Figure 18:**
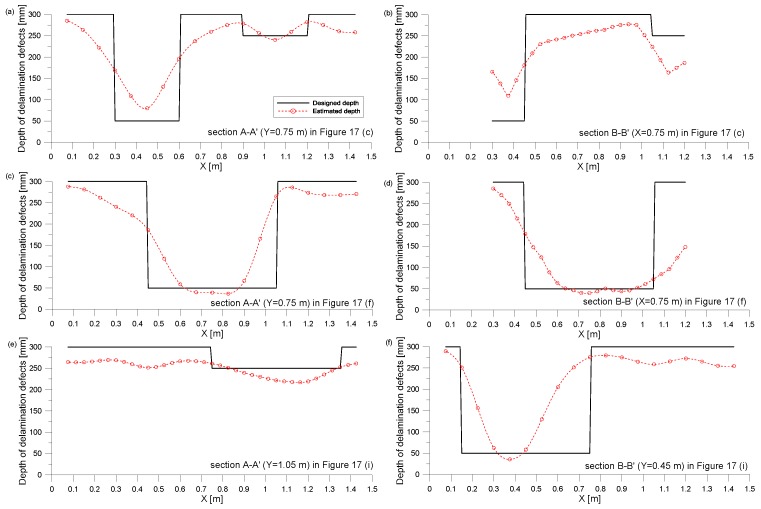
Comparison of estimated and as-built depth of delamination defects and thickness of solid concrete: (**a**) section A-A’ in Figure 17c; (**b**) section B-B’ in Figure 17c; (**c**) section A-A’ in Figure 17f; (**d**) section B-B’ in Figure 17f; (**e**) section A-A’ in Figure 17i; (**f**) section B-B’ in Figure 17i.

Figure 18 compares the estimated and as-built values of depths of delamination defects and thickness of plates. Overall, the estimated depths have a good agreement with the as-built depths. For the solid concrete and deep delamination defects, the mean of the estimated thickness is about 280 mm and 230 mm, respectively. Those values estimated by Equation (3) appears to underestimate the as-built depths. There are several reasons for underestimation [9]: (1) underestimation of phase velocity and (2) systematic errors due to near-field effect. More studies are still needed to accurately estimate the thickness (or depth) of relatively deep delamination defects. In contrast, the depth of the shallow delamination defects was obtained from the A0 Lamb wave analysis. It was observed that this method results in relatively accurate depth information of shallow delamination with relatively large areal extension.

## 6. Summary and Conclusions

Delamination defects in concrete slab specimens were evaluated by analyzing multi-channel elastic wave signals. In this study, a prototype eight-channel sensing device was developed for elastic wave measurements on the surface of concrete. Multi-channel elastic wave signals were converted to a frequency-phase velocity image by the phase-shift method. A data processing method was proposed to extract the two useful wave modes (A0 and S1 zero group velocity Lamb wave modes) from the dispersion image. The wave modes were used to characterize the depth and lateral dimensions of delamination defects in concrete. The validity of the data interpretation method was verified by comparison analysis between as-built and estimated results measured from concrete slab specimens with simulated delamination defects of various sizes and depths in the laboratory. The specific conclusions obtained from this study are summarized as follows:

(1) The depths of the delamination defects in concrete slab specimens were estimated by the two important wave modes: non-propagating waves (S1 ZGV Lamb mode) and propagating waves (A0 Lamb wave mode). The dominant non-propagating waves were effective for relatively deep delamination (250 mm in this study), while the propagating waves are valid for relatively shallow delamination (50 mm in this study). The estimated depths of delamination defects have a good agreement with the as-built depths in the simulated concrete slabs in this study. Test results show that the size of delamination defects have a great influence on the estimation errors, e_dS1ZGV_ or e_dA0_, of the depth of delamination defects from the S1 ZGV Lamb mode and A0 Lamb mode, respectively: lateral dimensions should be at least 300 mm to obtain relatively accurate depth estimation from the two important wave modes. According to experiences of the authors from the laboratory and field tests, the test method using the S1 ZGV Lamb mode can be applicable to the delamination defects with a depth greater than 150 mm; and the analysis of A0 Lamb wave mode to the shallow delamination defects with a depth less than 100 mm.
(2) It was demonstrated that the non-propagating waves associated with flexural vibration modes over relatively shallow delamination defects in concrete are effective for estimating the lateral dimensions of the defects. The estimated lateral dimension of the shallow delamination defects (DL1, 2, 5, and 6) in the concrete slab specimens in this study show a good agreement with the as-built sizes, with the estimation errors (e_c_) ranging from 4.6% to 13% without regard to the size of delamination defects considered in this study.
(3) Delamination defects in concrete slab specimens were visualized by mapping the three critical parameters, which include surface wave velocity, peak frequency, and depth of delamination defects. The delamination maps were effective for visualizing the areal extents and depths of delamination defects in concrete, which gives a way of estimating the damage volume in concrete.
(4) There are two important benefits of using the multi-channel elastic wave measurements for the evaluation of delamination defects in concrete. First, it enables us to simultaneously extract two important wave modes (A0 and S1 ZGV Lamb wave mode). Second, the fusion of data from two important wave modes could result in a more accurate and consistent evaluation of delamination defects in concrete.
(5) The results in this study were obtained from concrete slab specimens with simulated delamination defects in the laboratory. More studies are still needed to obtain more general conclusions of the feasibility of multi-channel elastic wave measurements.

## Figures and Tables

**Table 1:** Summary of results over delamination defects in concrete specimens.

ID	As-Built Dimensions [mm]			*f* [Hz]	*V_R_* [m/s]	Estimated Dimensions [mm]			Error [%]		
	c_x_	c_y_	d			$c^{\prime }$	$d_{A0}^{\prime }$	$d_{S1ZGV}^{\prime }$	e_c_	e_dA0_	e_dS1ZGV_
Solid	-	-	300	5800	1831	-	191	297	-	36.3	1.0
DL1	300	300	50	2100	1567	286	87	785	4.6	74.0	1470.0
DL2	150	150	50	4100	1589	167	106	421	11.3	112.0	742.0
DL3	300	300	250	8100	1729	-	143	213	-	42.8	14.8
DL4	150	150	250	4700	1825	-	136	367	-	45.6	46.8
DL5	600	600	50	2100	1408	522	47	822	13.0	6.0	1544.0
DL6	600	300	50	2300	1547	277	56	751	7.6	12.0	1402.0
DL7	600	300	250	6600	1834	-	175	261	-	30.0	4.4

**Table 2:** Summary of K-S test results and mean, standard deviation and coefficient of variation of data sets from three groups.

ID	K-S Test Results		Mean [m/s]	Standard Deviation [m/s]	COV [%]
	h	*p*-Value			
Group 1 over solid concrete	0	0.3040	1845	97.0	5.3
Group 2 over deep DL	0	0.6710	1850	74.1	4.1
Group 3 over shallow DL	0	0.6926	1582	169	10.7

Note: h is the result of the K-S test: h = 0 indicates not reject the null hypothesis (normal distribution) at the significant level of 5% in this study.

**Table 3:** Summary of K-S test results and mean, standard deviation and coefficient of variation of data sets from three groups.

ID		K-S Test Results		Mean [Hz]	Standard Deviation [Hz]	COV [%]
		h	*p*-Value			
Group 1		0	0.2550	6003	437	7.3
Group 2		0	0.8710	6445	760	11.8
Group 3	core	0	0.0843	3298	362	10.9
	perimeter	0	0.3435	5658	606	10.6

Note: h is the result of the K-S test: h = 0 indicates not reject the null hypothesis (normal distribution) at the significant level of 5% in this study.
